# Hemoperitoneum from Corpus Luteal Cyst Rupture: A Practical Approach in Emergency Room

**DOI:** 10.1155/2014/252657

**Published:** 2014-06-01

**Authors:** Valeria Fiaschetti, Aurora Ricci, Angela Lia Scarano, Valeria Liberto, Daniele Citraro, Silvia Arduini, Giuseppe Sorrenti, Giovanni Simonetti

**Affiliations:** ^1^Department of Diagnostic Imaging, Molecular Imaging, Interventional Radiology and Radiation Therapy, University Hospital Tor Vergata, Viale Oxford, 81-00133 Rome, Italy; ^2^Section of Gynecology and Obstetrics, Department of Biomedicine & Prevention, University Hospital Tor Vergata, Viale Oxford, 81-00133 Rome, Italy

## Abstract

Corpus luteum cyst rupture with consequent hemoperitoneum is a common disorder in women in their reproductive age. This condition should be promptly recognized and treated because a delayed diagnosis may significantly reduce women's fertility and intra-abdominal bleeding may be life-threatening. Many imaging modalities play a key role in the diagnosis of acute pelvic pain from gynecological causes. Ultrasound study (USS) is usually the first imaging technique for initial evaluation. USS is used to confirm or to exclude the presence of intraperitoneal fluid but it has some limitations in the identification of the bleeding source. Contrast-enhanced computed tomography (CT) is the imaging modality which could be used in the acute setting in order to recognize gynecological emergencies and to establish a correct management. Magnetic resonance imaging (MRI) nowadays is the most useful technique for studying the pelvis but its low availability and the long acquisition time of the images limit its usefulness in characterization of acute gynecological complications. We report a case of a young patient with hemoperitoneum from hemorrhagic corpus luteum correctly identified by transabdominal USS and contrast-enhanced CT.

## 1. Introduction

Acute pelvic pain in women of childbearing age is a common and frequent cause for admission to emergency room (ER), necessitating emergent medical evaluation especially when it is due to hemoperitoneum [1].

In this scenario, the wide range of differential diagnoses that must be considered when assessing abdominal pain represents an issue for the clinical approach.

Sometimes it can be difficult to distinguish gynecological from gastrointestinal and urinary tract emergencies because of overlapping symptoms and signs. Various imaging modalities in association with clinical findings play an important role in the characterization of the cause of pain [2, 3].

Early diagnosis is necessary to preserve the reproductive systems and the life of the patient in severe cases. Hemoperitoneum may occur in the context of various gynecological emergencies; in some cases it could be a complication of a ruptured hemorrhagic corpus luteum [3–5].

Suspecting gynecological disease in young women the use of X-ray radiation and computed tomography (CT) is not recommended due to the risks associated with irradiating the pelvis. Ultrasound study (USS) and magnetic resonance imaging (MRI) are the preferred imaging investigations in pelvic diseases; however, in gynaecological emergencies, when a simple and rapid assessment of the patient's condition is required and once pregnancy is excluded, the CT is the investigation of choice [4].

We describe a case of hemoperitoneum from a ruptured hemorrhagic corpus luteum in an adolescent woman in which the use of emergent CT in the ER was necessary in order to obtain the correct diagnosis.

## 2. Case Presentation

A 16-year-old adolescent female presented to the ER with sudden onset abdominal pain, lasting for six hours. Her last menstrual period was 18 days back. The patient did not have previous diseases or surgery.

On physical examination she presented tachycardia (pulse rate 115 beats per minute), tachypnea (respiratory rate 22 breaths per minute), and hyperpyrexia (37.8°C) with abdominal distention and severe rigidity on the lower quadrants on palpation. Blood pressure (BP) was 85/60 mmHg and she was anxious but alert and oriented.

Laboratory investigations showed mild leukocytosis, slight elevation of C-reactive protein (CRP) (192 mg/L), and discrete anemia (hemoglobin 10 g/dL). Urine output was 26 cc/h and serum; *β*-human chorionic gonadotropin (*β*hCG) level was below 4 UI/L. Gynecological examination excluded abnormalities of the external genital structures in a virgin patient.

**Figure 1:**
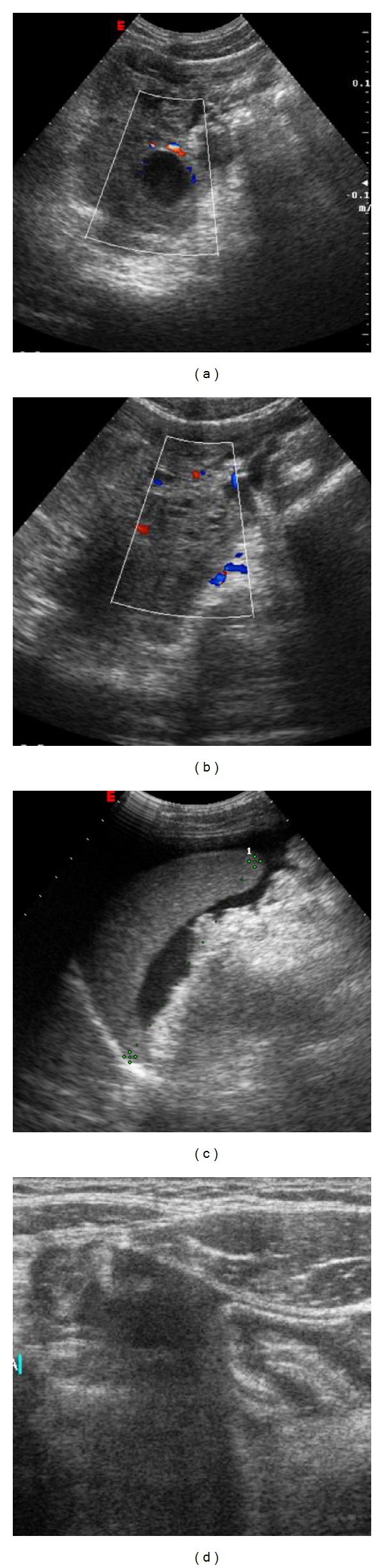
Transabdominal US shows on the right adnexal area a regular cyst with internal echoes and peripheral vascularization on Doppler evaluation (a). Free fluid into the pouch of Douglas (b) and around the spleen (c). (d) Ultrasonographic scanning with linear probe shows free fluid containing low level echoes in the right iliac fossa. No signs of appendicitis are appreciable.

Transabdominal USS revealed a moderate amount of fluid in the abdomen and in the pouch of Douglas and a complex right adnexal mass with signs of peripheral vascularization (Figure 1).

An abdominal CT study was indicated and performed using a 64-row scanner (LightSpeed VCT, General Electric Medical System, Milwaukee, WI, USA), before and after the injection of iodinate contrast medium (Iomeron 350 mg/mL, Bracco Imaging, Milan, Italy).

**Figure 2:**
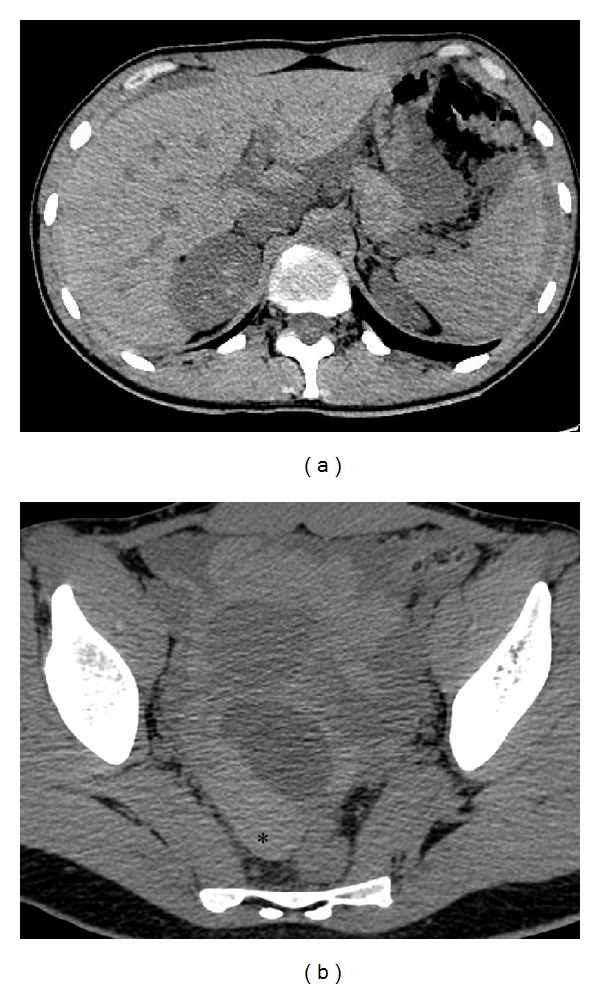
Unenhanced CT axial images (a, b) show diffuse peritoneal effusion in abdomen with different attenuation values that progressively increase from the upper abdomen (a) to the pelvis (b) where it becomes strongly hyperdense (60–65 HU,*) for the presence of blood component. (b) Low-density cyst is appreciable in the right adnexal area.

**Figure 3:**
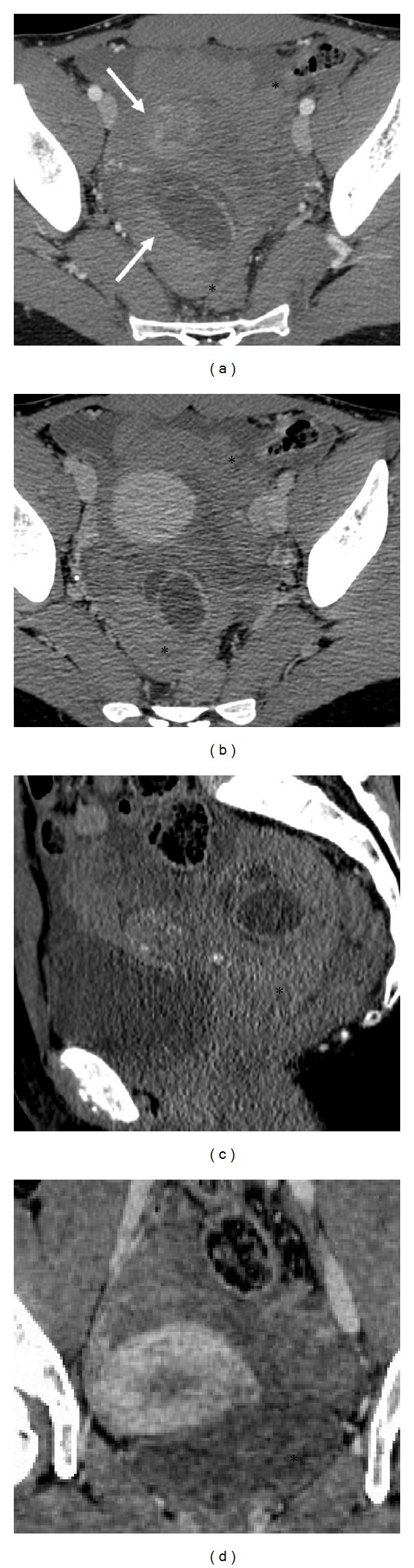
Dynamic contrast-enhancement CT scan. After injection of contrast medium pelvic CT images ((a, b) axial image; (c) sagittal image; (d) coronal image) show thickening and contrast-enhanced bilobate cystic wall in the right adnexa. The early arterial phase shows cloud-like extravasation from this lesion indicating active bleeding (arrows). Dynamic CT scan confirms diffuse hemoperitoneum (*).

The unenhanced CT scan (Figure 2) showed a low-attenuation fluid within the peritoneum surrounding spleen and part of liver with extension to the pouch of Douglas and to the periuterine area, where a round hypodense image was detected. The fluid showed progressively increasing attenuation values from the upper abdomen to the pelvis and it appeared clearly hyperdense in the pouch of Douglas (60–65 Hounsfield units, HU). These findings were suggestive for the presence of hemoperitoneum. A dynamic CT scan revealed thickened cysts with enhancing walls, such as a corpus luteum cyst. The arterial phase showed a cloud-like extravasation from the cyst, indicating the presence of active bleeding (Figure 3). The CT study excluded other diseases of the intra-abdominal organs, such as the uterus and left adnexae.

After fluid resuscitation, the patient became haemodynamically stable and the subsequent emergent laparoscopy confirmed a bleeding corpus luteum as the cause of the hemoperitoneum.

## 3. Discussion

Spontaneous hemoperitoneum may occur in various gynecological emergencies. The most common gynecological causes of spontaneous hemoperitoneum in women of childbearing age are ectopic pregnancy and ruptured corpus luteal cyst. More uncommon causes are uterine rupture, endometriosis, and ruptured hydropyosalpinx [5].

Corpus luteum is a functional cyst developing in the luteal phase of the ovarian cycle which regresses spontaneously in corpus albicans when pregnancy does not occur [6]. Being a thin-walled vascular structure corpus luteum is prone to hemorrhage even if bleeding is usually contained inside the cyst [2]. Corpus luteum cyst-wall rupture is a rare complication that occurs most frequently in women in their reproductive age but it is relatively uncommon in early adolescence [6, 7]. When bleeding occurs, hemorrhage may spread into the peritoneal cavity causing hemoperitoneum [2].

The diagnosis of ruptured corpus luteal cyst is based on a high historical suspicion (the patient generally is in the luteal phase of the ovarian cycle), clinical features, and laboratory tests. The latter often show anemia, raised CRP, and mild leukocytosis. These signs and symptoms are similar to gastrointestinal tract diseases. Patients may present a wide range of clinical signs, from no signs to severe peritoneal irritation which can be confused with, for example, acute appendicitis. The evaluation of serum *β*hCG-levels is necessary to differentiate ruptured corpus luteal cyst from ruptured ectopic pregnancy, which may have a similar presentation [4, 8]. A persistent corpus luteum may be associated with delayed menstrual cycle. Occurrence of a corpus luteum rupture may be indicative of the presence of an intrauterine pregnancy. Therefore, a ruptured corpus luteum cyst rupture should be considered even in the presence of a positive pregnancy test [7]. Various imaging modalities play an important role in diagnosing the ruptured corpus luteum cyst. Usually, USS is the first imaging modality due to its high sensitivity and fast and easy access. On the other hand, it can be difficult to localize the site of the disease and bleeding [3, 4, 9]. Bennett in ruptured corpus luteal cyst USS may reveal a complex cyst, with a rim of increased echogenicity surrounding the cystic component in the adnexal area, associated with free hypoechoic fluid in the peritoneal cavity (hemoperitoneum). Free hypoechoic fluid may contain focal collections of higher echogenicity (e.g., clotted blood) in the pelvis [2, 4]. Doppler USS may demonstrate the vascularized wall [4, 10].

Although MRI is the most adequate technique for the pelvic evaluation, thanks to its high-soft-tissue contrast capability, it is not usually used in the acute setting due to its considerably long acquisition time, limited availability, and high costs [3, 4].

For these reasons, MRI has limited availability in emergency department and it is not currently included in the American College of Radiology Appropriateness Criteria [11].

Therefore, in the ER gynecological diseases are frequently detected with CT examination [2, 4, 5]. The spectrum of attenuation values of abdominal fluid will direct the diagnosis of hemoperitoneum. Body fluids, with a density similar to water, have attenuation values in the range of 0–15 HU. Blood, which has a higher density than other body fluids, has attenuation values of 30–45 HU. Examination of attenuation values will permit estimating the time from bleeding, the diffusion, and location of the hemorrhage [3]. Peritoneal fluid typically shows higher attenuation values near the source of bleeding; these parameters progressively increase from the upper abdomen to the parietocolic gutters, where the pelvis becomes hyperdense (60–65 HU) [3]. The area with the highest attenuation values is defined as “sentinel clot” and it indicates the source of bleeding [4].

On CT examination, corpus luteum usually appears like a well-circumscribed unilocular adnexal lesion, rarely bilocular. The cyst walls appear slightly thickened (<3 mm) and show a characteristic inhomogeneous contrast enhancement after administration of contrast medium due to increased vascularity. The cystic content is mixed with a high attenuation component (45–100 HU) and in some cases it presents a “fluid-fluid hematocrit” level [4].

Contrast-enhanced CT may be helpful in excluding other intra-abdominal diseases (e.g., ruptured hepatic adenoma) that can cause hemoperitoneum in the young female patient [4, 9].

For the management of hemoperitoneum a standard algorithm is not reported in literature. Historically the treatment of corpus luteum hemorrhage was exclusively surgical, while nowadays it can be managed with a conservative approach. In either case the treatment targets at preserving ovarian function as well as at eliminating the source of bleeding [12].

In particular, the conservative approach is the first-choice treatment when the patient is hemodynamically stable (systolic BP > 90 mmHg) with hemoglobin values that keep being constant over 4–6 hours of monitoring [13]. In case of unstable vital signs the patient undergoes surgical treatment. Laparoscopy represents the first minimally invasive approach [14] that can eventually be converted to laparotomy in case of failure or of unstable vital signs such as important hemoglobin decrease over 4–6 hours (~2 g/dL) with increasing hemoperitoneum on follow-up imaging studies [13, 15].

## 4. Conclusion

In summary, USS is a helpful imaging modality in diagnosing some gynecological emergencies in the ER but may not detect the entire range of the disease, whereas contrast-enhanced CT of the abdomen is recommended in the acute ER setting to diagnose gynecological emergencies and to initiate correct management in a timely fashion.
